# Hallucinations in an Elderly Patient with Severe Visual Impairment

**DOI:** 10.7759/cureus.3592

**Published:** 2018-11-14

**Authors:** Barjinder S Buttar, Alan T Kaell

**Affiliations:** 1 Internal Medicine, Northwell Health Mather Hospital, Port Jefferson, USA

**Keywords:** visual hallucinations, charles bonnet syndrome, anton-babinski syndrome, geriatrics, visual impairment, psychiatry, internal medicine, general practice, preventive medicine, neurology

## Abstract

Vivid visual hallucinations are common in the geriatric population and can be due to a number of causes such as dementia, delirium, stroke, adverse effects of medication, or ocular disease. We will examine an interesting patient case report, which allowed us to explore two lesser-known causes of these types of hallucinations: Charles Bonnet Syndrome and Anton-Babinski Syndrome. Treatment of these syndromes focuses on supportive care as well as extensive education for the patient and family concerning the benign nature of the visual hallucinations. Many patients, however, end up undergoing extensive diagnostic studies and treatments that are not necessary. This occurs as a result of a lack of education when it comes to the diagnosis and management of these conditions. By raising awareness among healthcare providers, we can prevent unnecessary and potentially harmful workups and treatments for patients suffering from these syndromes.

## Introduction

Charles Bonnet Syndrome is a type of psychophysical visual disturbance seen in the geriatric population among individuals who have experienced significant vision loss. Patients will experience vivid, complex, recurrent visual hallucinations, commonly of faces, animals, or cartoons. The diagnostic criteria for this syndrome include severe visual impairment, visual hallucinations, partially or fully intact insight, no evidence of brain disease or other psychotic disorder, and no other senses affected [1].

Anton-Babinski Syndrome is a rare condition seen in patients following a stroke or significant trauma to the head. It is characterized by visual anosognosia, or denial of vision loss, which is associated with confabulation in the setting of vision loss and cortical blindness. Confabulations are a defining characteristic of this syndrome. The damaged visual areas are disconnected from functioning areas, such as speech and language. Without input, speech areas will confabulate a response [2].

## Case presentation

**Figure 1:**
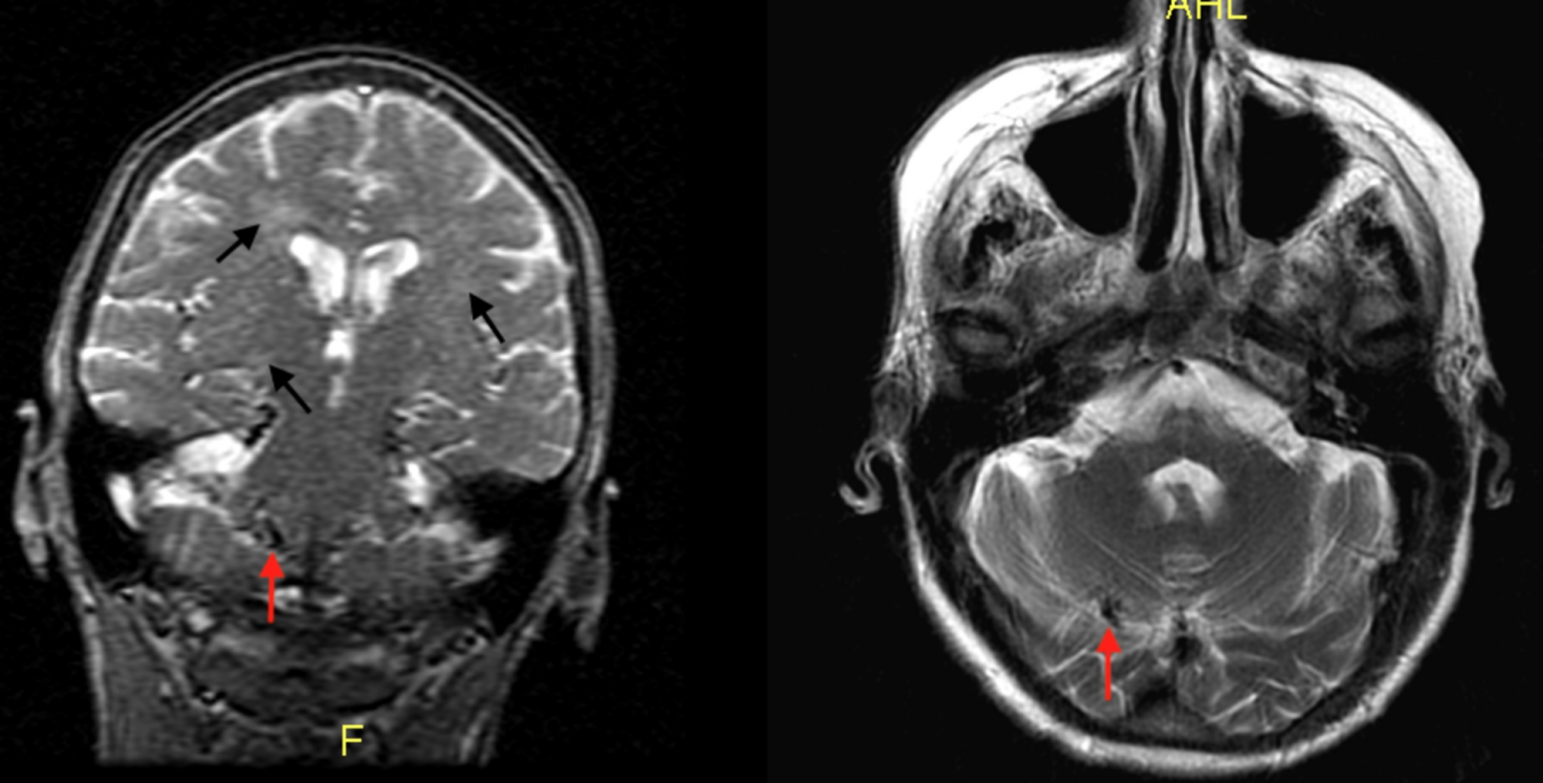
Imaging of the Brain Without Contrast There is no evidence of intracranial hemorrhage. Global cerebral atrophy and a significant chronic small vessel ischemic disease are appreciated (black arrows). There is a stable, small focus in the superior right cerebellar hemisphere, which is indicative of an arteriovenous malformation (red arrow).

This case report will focus on an 80-year-old female with a history of multiple transient ischemic attacks, cerebral arteriovenous malformation, and deteriorating vision with complete loss of vision in the left eye. She presented to the emergency room with a chief complaint of seeing vivid organisms with tentacles in her food and stool. The patient was well-kept, organized, alert, and oriented to person, place, time, and situation. She provided a very detailed history, stating that the vivid hallucinations started eight weeks ago. She would see them in her eggs when she ate breakfast and when she drank water or juice. She had seen the hallucinations in her stool as well. The patient meticulously collected samples of her food and stool in containers and brought them to an urgent care center for evaluation. From the urgent care center, she was sent directly to the emergency room. During her admission, the patient stated she understands and is fully aware of the fact that the tentacles are visual hallucinations. Her symptoms have been a significant source of worsening stress and anxiety over the past few weeks. Her vitals, labs, blood, and urine cultures were all normal. Her urine toxicology screen was negative. A computed tomography (CT) scan of the head showed no acute intracranial findings and magnetic resonance imaging (MRI) of the brain was negative for any acute changes, as seen in Figure 1.

Psychiatry was consulted, and our team determined that her insight was fully intact. Her symptoms could not be attributed to an acute psychotic episode. We then began to discuss the possibility of Charles Bonnet Syndrome and Anton-Babinski Syndrome as potential differential diagnoses. Charles Bonnet Syndrome was confirmed because the patient fit all the relevant diagnostic criteria. This included significant visual impairment, persistent visual hallucinations, fully intact insight, no evidence of acute psychosis, no evidence of stroke or hemorrhage, and no other senses other than her vision were affected. Anton-Babinski Syndrome was ruled out because this patient did not have a recent stroke or trauma to the head and there were no definitive confabulations with denial of vision loss. Once the diagnosis was made, the patient and her family were educated on the benign nature of her visual hallucinations. Supportive care was provided and the patient was discharged home.

## Discussion

**Figure 2:**
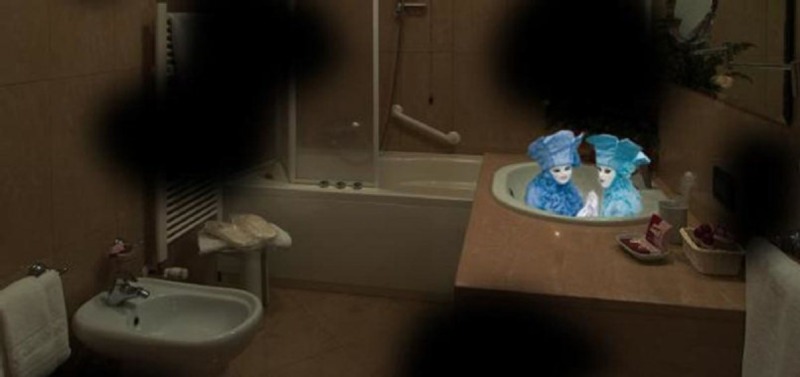
Lilliputian Visions in a Patient Diagnosed with Charles Bonnet Syndrome Lilliputian visual hallucinations, or little people, are commonly seen in patients suffering from Charles Bonnet Syndrome. The miniature people are often interacting with one another and are brightly dressed [4].

**Figure 3:**
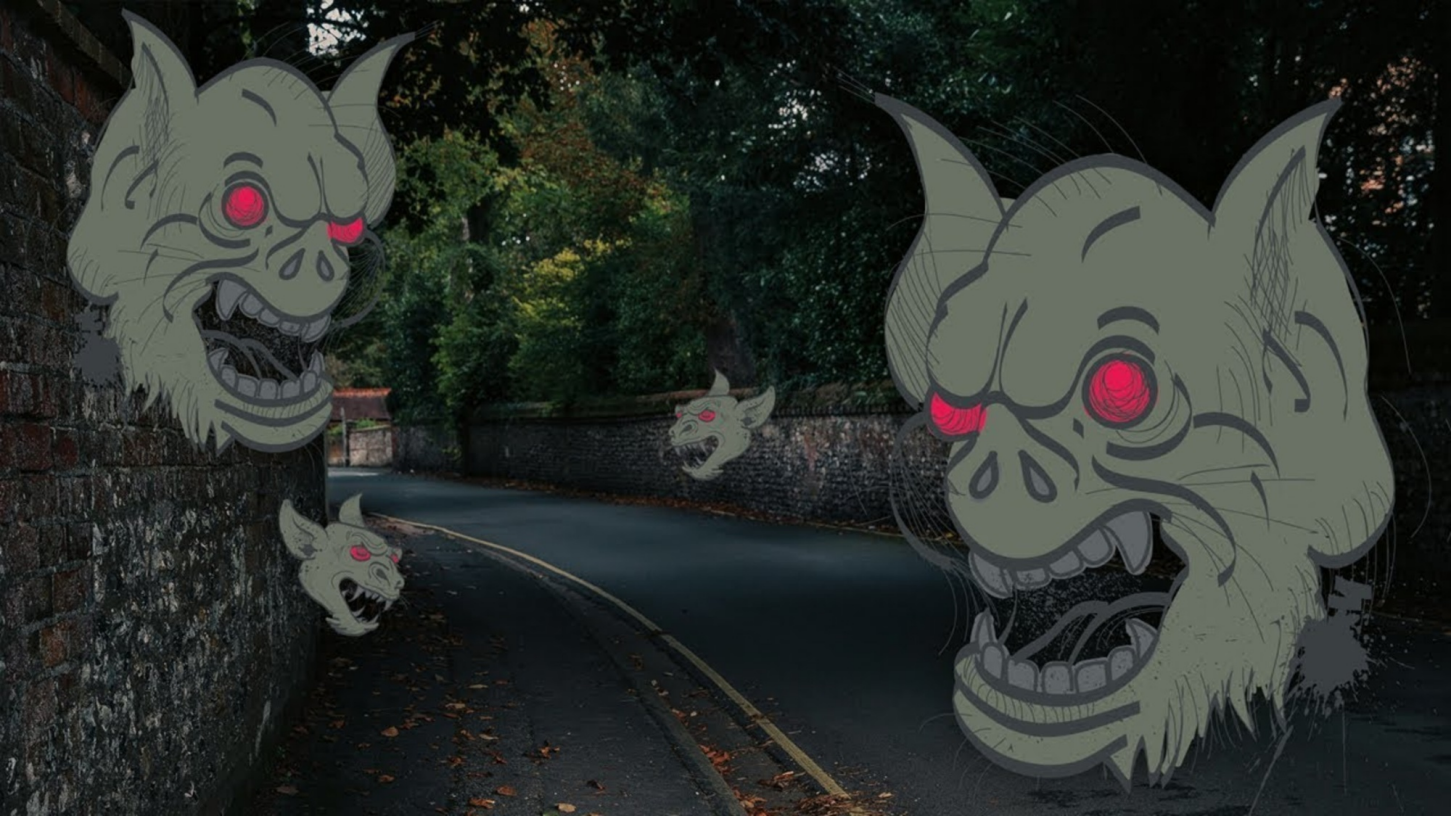
Ominous Visions in a Patient Diagnosed with Charles Bonnet Syndrome Scary visual hallucinations are rare in patients with Charles Bonnet Syndrome but do occur. These visions can present, for example, as large and dark gargoyle heads [5].

The prevalence of both these syndromes in the geriatric population who suffer from vision impairment varies from 10 to 15 percent with Anton-Babinski Syndrome being much less common. Patients who suffer from these vivid, visual hallucinations tend to under-report symptoms to healthcare providers due to a fear of being diagnosed with a mental illness [3]. Figure 2 and Figure 3 show examples of classic, vivid hallucinations seen by patients suffering from Charles Bonnet Syndrome. Figure 2 specifically shows Lilliputian visions or little people who are often brightly dressed. Figure 3 shows more unpleasant and stress-inducing visions of large gargoyle heads.

**Figure 4:**
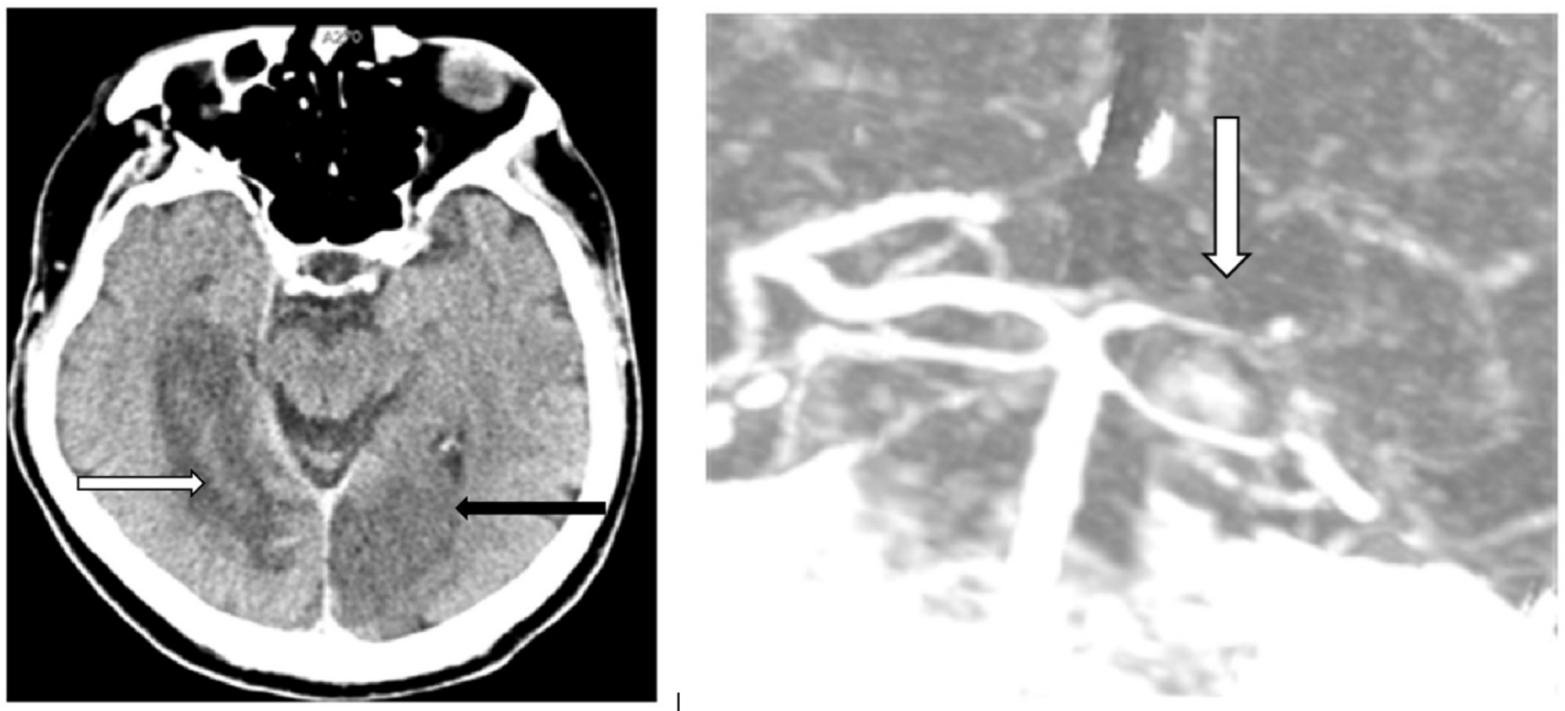
Patient Diagnosed with Anton-Babinski Syndrome Left: Axial computed tomography (CT) brain showing a subacute infarction in the right posterior cerebral artery (white arrow) and a new acute cerebral infarction in the left posterior cerebral artery (black arrow). Right: CT angiography showing the gracile flow of the right posterior cerebral artery (white arrow).

When diagnosing a patient with Anton-Babinski Syndrome, the main differentiating characteristics are the presence of an acute stroke or trauma to the head, visual anosognosia, and confabulations. Zukić et al. presented a rare case of a patient who had successive bilateral occipital lobe infarcts due to massive stenosis of the arteries of the head and neck. As a result of this, he was blind and would confabulate to fill in the missing sensory input [2]. Figure 4 shows a CT of the brain of this patient with bilateral right and left posterior cerebral artery infarctions [2]. Figure 4 also shows a CT angiogram of the brain with stenosis of the left subclavian artery [2].

The stress and anxiety that builds up as a result of these hallucinations take a huge toll on patients. When they do report symptoms, the immediate response of healthcare providers is to do a full workup, including extensive lab work, imaging, and a psychiatric evaluation. Subjecting patients to this stressful environment is harsh and potentially dangerous. An extensive workup could lead to patients being misdiagnosed and started on unnecessary medications, which can have dangerous consequences.

Healthcare providers need to be aware of these syndromes and should include them in the differential diagnosis of elderly patients with significant visual impairment presenting with visual hallucinations. This will help to greatly reduce extensive workups that are not required. Healthcare providers also need to be aware of how to properly treat these patients. The most effective treatment option is supportive care. The main goal is to assure patients, their caregivers, and their families that the hallucinations stem from their visual impairment and are not indicative of mental illness. It is important to encourage them to participate in regular social contact and take part in activities that actively engage the mind. Treating any serious ophthalmologic diseases patients may suffer from, optimizing their vision, improving lighting, and minimizing glare can help reduce the hallucinations as well. If supportive measures are not effective, we can then turn to pharmacologic interventions, such as selective serotonin reuptake inhibitors, antipsychotics, anticonvulsants, and cholinesterase Inhibitors [6].

## Conclusions

As healthcare providers, we need to understand and recognize these conditions so that these patients can be properly managed. In a study by Gilmour et al., only nine percent of individuals who had experienced Charles Bonnet Syndrome sought medical advice. Disappointingly, only half of them received a detailed explanation of the syndrome, how it presents, and how to treat it. Studies have shown that there is a strong trend associated with the quality of information provided by medical professionals and whether a negative outcome was experienced. When patients suffering from Charles Bonnet Syndrome are not given a clear explanation, the majority will go on to experience negative outcomes with persistent hallucinations. On the other hand, when patients with the syndrome are properly educated, they improve greatly over time. Many of them will even go on to experience complete resolution of the hallucinations. When it comes to awareness among healthcare providers, it has been found that one-third of medical professionals were either uncertain or unaware of Charles Bonnet Syndrome and how to diagnose it. By simply educating healthcare providers, we can do so much to reduce the unnecessary suffering of these patients.
